# Exploring emotional patterns in social media through NLP models to unravel mental health insights

**DOI:** 10.1049/htl2.12096

**Published:** 2025-01-09

**Authors:** Nisha P. Shetty, Yashraj Singh, Veeraj Hegde, D. Cenitta, Dhruthi K

**Affiliations:** ^1^ Department of Information and Communication Technology Manipal Institute of Technology Manipal Academy of Higher Education Manipal Karnataka India; ^2^ Department of Computer Science and Engineering Manipal Institute of Technology Manipal Academy of Higher Education Manipal Karnataka India; ^3^ Department of SWASTHAVRITTA and YOGA Yenepoya Ayurveda Medical College & Hospital Manjanade Karnataka India

**Keywords:** correlation methods, data mining, health hazards, medical information systems

## Abstract

This study aimed to develop an advanced ensemble approach for automated classification of mental health disorders in social media posts. The research question was: can an ensemble of fine‐tuned transformer models (XLNet, RoBERTa, and ELECTRA) with Bayesian hyperparameter optimization improve the accuracy of mental health disorder classification in social media text. Three transformer models (XLNet, RoBERTa, and ELECTRA) were fine‐tuned on a dataset of social media posts labelled with 15 distinct mental health disorders. Bayesian optimization was employed for hyperparameter tuning, optimizing learning rate, number of epochs, gradient accumulation steps, and weight decay. A voting ensemble approach was then implemented to combine the predictions of the individual models. The proposed voting ensemble achieved the highest accuracy of 0.780, outperforming the individual models: XLNet (0.767), RoBERTa (0.775), and ELECTRA (0.755). The proposed ensemble approach, integrating XLNet, RoBERTa, and ELECTRA with Bayesian hyperparameter optimization, demonstrated improved accuracy in classifying mental health disorders from social media posts. This method shows promise for enhancing digital mental health research and potentially aiding in early detection and intervention strategies. Future work should focus on expanding the dataset, exploring additional ensemble techniques, and investigating the model's performance across different social media platforms and languages.

## Graphical abstract

The study aimed to enhance the accuracy of classifying mental health disorders in social media posts by developing an ensemble of fine‐tuned transformer models (XLNet, RoBERTa, and ELECTRA) using Bayesian hyperparameter optimization. Three transformer models (XLNet, RoBERTa, and ELECTRA) were fine‐tuned on social media posts labelled with 15 mental health disorders. Bayesian Optimization was used to tune hyperparameters, and a voting ensemble approach was applied to aggregate model predictions. The ensemble approach achieved the highest accuracy of 0.780, outperforming individual models and showing a 1.3% improvement over the best‐performing model, XLNet. This demonstrates the potential of the method for digital mental health research and early detection strategies. Future research should expand the dataset, explore new ensemble techniques, and test model performance on different platforms and languages.

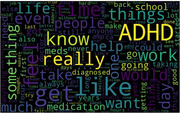

## 1 INTRODUCTION

Mental health disorders constitute a significant global health concern. In 2019, one in eight people would have been affected by a mental disorder at some point in their lives, with approximately 970 million people suffering from such conditions [1].

Mental health disorders can severely impact an individual's quality of life, often leading to physical health issues, social impairment, and increased economic burden. Furthermore, early detection and intervention significantly improve treatment outcomes and reduce the severity and duration of these disorders. Thus, there is a critical need for timely and effective means of identification.

In the modern world, social media platforms have become integrated with our daily lives and constitute a rich source of information about individuals’ emotional states. These platforms offer a unique perspective into individuals’ emotions, attitudes, and behaviours in real‐time, thus providing unprecedented opportunities for mental health research. Recent research suggests that analysis of social media data can reveal subtle clues about mental health disorders before individuals may even be aware of their condition, facilitating early intervention [2].

Natural language processing (NLP), a subfield of artificial intelligence (AI), offers valuable tools for mining such insights from the vast and growing volume of social media data. NLP techniques enable machines to understand and analyse human language, and when applied to social media data, they can detect patterns and trends in users’ emotional states. Specifically, emotional analysis, a key application of NLP, can assess the sentiment and emotions expressed in the text, thus providing critical insight into mental health conditions.

Given this background, our study seeks to leverage these opportunities by harnessing the power of advanced machine‐learning techniques. We adopt an ensemble approach, utilizing three transformer‐based models: XLNet, RoBERTa, and ELECTRA—that have shown robust performance in various NLP tasks. These models are fine‐tuned on a rich corpus of social media data, specifically the Reddit mental health dataset, to classify 15 different mental health disorders such as alcoholism, addiction, autism, ADHD, anxiety, bipolar disorder, borderline personality disorder (BPD), various eating disorders, depression, health anxiety, loneliness, post‐traumatic stress disorder (PTSD), schizophrenia, social anxiety, and suicide watch.

An integral part of our methodology is the optimization of model hyperparameters to improve performance. We utilize Bayesian optimization with the Optuna framework to tune crucial parameters including the learning rate, number of training epochs, gradient accumulation steps, and weight decay. This optimization process helps ensure that our models are not only effectively learning the nuanced language patterns associated with different mental health disorders but also doing so in a computationally efficient way [3].

Our research has the potential to contribute significantly to the burgeoning field of digital mental health. By providing a sophisticated method for the automated classification of mental health disorders in a social media text, we aim to support mental health professionals, researchers, and social media platforms in understanding and responding to mental health discussions online. This paper outlines our methodology, the performance of the individual models and their ensemble, and the potential implications and future research directions in this area.

## 2 MOTIVATION AND SCOPE OF THE STUDY

### 2.1 Motivation of the study

The global burden of mental health disorders is staggering, affecting hundreds of millions of people worldwide. With the advent of social media platforms, there has been a transformation in how we communicate, and this extends to sharing experiences related to mental health. Notably, these platforms have become particularly crucial for Generation Z, those born between 1997 and 2012, who frequently turn to social media to express their emotions, fears, and hopes.

A recent study revealed that Generation Z is more likely to report mental health concerns than previous generations, and they often use social media as a platform to communicate their struggles. For instance, research conducted on a sample of 512 students showed how social media amplified negative emotions resulting in worse mental health [4]. The openness of Generation Z in discussing mental health online, coupled with the vast amount of data available, underscores the necessity and timeliness of our research.

As these platforms become increasingly influential in shaping public discourse, they also offer a treasure trove of data for mental health research. This data, while voluminous and intricate, presents a unique opportunity to understand mental health trends and challenges at a population level, especially among the younger generation. However, the manual analysis of such a large amount of data is nearly impossible due to its sheer scale. Traditional automated methods, on the other hand, often struggle to effectively capture the nuanced language associated with mental health discussions. It is this gap we aim to bridge, motivated by the potential of deep learning models to analyse and classify mental health‐related content on social media platforms effectively.

In doing so, we strive to provide an advanced tool that can assist researchers, clinicians, and policymakers in monitoring mental health trends, identifying emerging concerns, and shaping timely interventions. The importance of such a tool becomes even more significant in light of the communication preferences of Generation Z and their increasing inclination towards expressing their mental health challenges on social media platforms.

### 2.2 Scope of the study

Our study is intricately focused on the exploration and application of three leading transformer‐based models: XLNet, RoBERTa, and ELECTRA. Each of these models has demonstrated substantial efficacy in the realm of natural language processing tasks, thereby making them suitable candidates for our research. Integral to our methodology is an exhaustive hyperparameter tuning process utilizing Bayesian optimization within the Optuna framework. This process is crucial as it aids in enhancing the performance of these sophisticated models, thereby ensuring optimal results in terms of classification accuracy. Our primary aim is to scrutinize the efficacy of these models, both individually and in their collective capacity when amalgamated in an ensemble approach.

Despite its targeted focus, it is essential to clarify the limits of our research. This study does not venture into the practical implementation of the models in a real‐ world setting. We do not delve into the development of a user‐friendly application for deployment in real‐world scenarios. Our research remains primarily academic, striving to push the boundaries of our understanding of mental health classification through machine learning techniques.

Further, our analysis maintains a wider lens, concentrating on overarching trends and patterns associated with different mental health disorders represented in social media discourse. We refrain from drilling down to the individual user level, thereby preserving privacy and emphasizing the broader scope of population‐level mental health trends.

Additionally, the research is grounded in the analysis of English language social media data. We recognize the vast array of languages and communication styles present in the online world; however, for this study, our focus is confined to the English language, as it constitutes a significant portion of online communication, especially on the platforms of interest.

While these boundaries define the immediate scope of our research, it is imperative to note that the potential application of our methodology and findings extends far beyond these confines, especially in the broader field of natural language processing (NLP). The techniques and insights gained from our study, such as the successful application of transformer‐based models and the detailed hyperparameter tuning process, can offer significant contributions to other NLP tasks. This includes, but is not limited to, sentiment analysis, topic modelling, spam detection, and more.

Moreover, our rigorous hyperparameter optimization process, given its generic nature, could be employed to enhance the performance of a wide array of NLP models in various contexts. This opens up new avenues for improving model performance in a myriad of applications, making our study a valuable resource for future research in the NLP domain. Consequently, while our research is anchored in the realm of digital mental health, the ripple effects of our findings can be perceived in a far wider context, contributing substantially to the expansive and evolving landscape of natural language processing.

## 3 RELATED WORKS

Pioneering research in the domain of natural language processing (NLP) has underscored the potential of transformer models such as BERT, GPT‐2, and XLNet. A study by Hemmatirad et al. aimed to classify into two groups: positive and control [5]. While the study contributed to the field, it primarily centred around binary classification, leaving an open avenue for more comprehensive multi‐class classification, as proposed by our study.

Similarly, subsequent research efforts have utilized transformer models for mental health analysis, albeit largely restricting their scope to a specific range of disorders or limiting the application to a single model. This gap in the existing literature motivates our work, which amalgamates the strengths of various transformer models to create a more comprehensive and accurate classification system.

A breakthrough in mental health analysis arrived with the advent of hybrid models, as demonstrated in the research work by Jos´e Alberto et al. This innovative approach fused the capabilities of the BERT model with traditional machine learning algorithms [6]. Inspired by this concept, our study attempts to extend the hybrid model approach, integrating multiple transformer models into an ensemble to enhance overall performance.

With the ongoing evolution of machine learning models, there is a pressing need for effective optimization strategies. A study addressing this need by Navaux et al., highlights the potential of Bayesian optimization for hyperparameter tuning [7]. Motivated by this revelation, our study incorporates Bayesian optimization to fine‐tune our model. Similarly, the research work by Nakamura and Hong introduced the technique of adaptive weight decay, contributing to improved validation accuracy [8]. This strategic approach inspired us to incorporate dynamic weight decay during our model training phase.

The literature enclosed below provides an indept exploration of current work done for depression detection with more employs transformers, machine‐deep learning and ensemble techniques. Figuerêdo et al. in their study [9] tried to advance the early detection of depression from Reddit posts by presenting an architecture containing a fusion of various word embedding models. Their study incorporated both early and late fusion approaches. Additionally, to emphasize the importance of emoticons 2 sets of experiments; one discarding emoticons and the other mapping the emoticons to suitable interpretations were presented. The authors advocate the implementation of contextualized BERT‐based models as an advancement in this field to infer better by learning inherent characteristics in the post. This has been incorporated in our study. Additionally, the authors state that the use of pre‐trained transfer learning models can improve the efficacy of the work. The use of reinforcement learning for the selection of depressive posts from social networks is also proposed.

Tavchioski et al. [10] explored combinations of BERT, RoBERTA, BERTweet, and mentalBERT through averaging and Bayesian‐based Ensembles to classify texts conveying depressions. The superior performance exhibited by mentalBERT and BERTweet implored the efficacy of using domain‐based transformer models. As a future scope, the authors suggested using ensembles of other BERT‐based models as incorporated in our work. Another major limitation of the study was that the model missed important details when it encountered longer posts because it abridged the input text.

The DSM‐V depression indicator scale and the text‐inferring skills of LLMs were merged in a novel depression diagnosis system dubbed DORIS in their work by Lan et al. [11]. High emotional intensity postings were filtered using text embedding models, which also summarised the messages into mood courses for the user. They underwent further vectorization to provide representations of every mood course. Gradient Boosting Trees were utilized for the categorization purpose. Furthermore, the authors achieved great explainability by developing a thorough explanation of the system's judgment utilizing the exegesis of high‐risk texts and depiction of mood history generated throughout the model's operation. The major study limitations as quoted by the authors are credence on a single data set and a lone focus on depression without analysing other comorbidities.

Shetty et al. [12] compared the performance of LSTM against the CNN classifier in detecting depression in Twitter posts. The authors also compared the model performances against different machine learning and vectorizer pairs. The main limitation observed the by authors was the limited data size. They plan to employ depression‐recognizing medical indicators like DSM‐V to augment their work in the future.

Le‐Hinh et al. [13] used an averaging‐based ensemble approach by combining multiple pre‐trained models such as BERT, XLNET, and RoBERTa to detect depressive sentiments in pre‐processed Reddit posts. Additionally, they utilized a domain‐specific variant of RoBERTa called DepRoBERTa to categorize the severity of depression. However, the authors did not optimize the hyperparameters of the models, which the current study has done, resulting in improved model performance.

To tackle the problems of overfitting, restricted generalization, and data complexity, the authors Ogunleye et al. [14] proposed using stacked ensemble machine learning models with sentiment indicators and SBERT to predict depression. Afinn was utilized to conduct sentiment analysis. A significant constraint noted was the absence of an emotion analysis module within their work. Their approach may also be improved by adding explainability to the black box SBERT models to obtain insights into the weight variations during training.

The researchers Rizwan et al. [15] fine‐tuned small transformer models such as electra small generator (ESG), electra Small discriminator (ESD), XtremeDistil‐L6 (XDL), and Albert Base V2 (ABV) to classify the intensity of depression in tweets. These fine‐tuned and optimized small transformer models showed similar performance to DistilBERT. The researchers proposed a weighted ensemble with soft voting as a future improvement, which we have implemented in our study. The main limitation was that the models were not suitable for predicting depression intensity in longer text snippets like Reddit posts, as they were designed for short tweets.

The authors Liu and Shi [16] used both user‐generated posts and posting behaviour to identify depressive users on social media, in order to address the issue of imbalanced data leading to low recognition rates. They pre‐processed the data on user messages and posting behaviour, and then used a combination of recursive elimination, extreme random trees, and mutual information to select the optimal features. Additionally, they developed a stacking ensemble of machine‐learning algorithms for effective categorization. However, the study may not be representative of the general population, and it did not account for comorbidities and associated mental disorders with depression due to a lack of clinical data. The authors also suggested investigating the incorporation of innovative learning approaches to see if changing variables or parameters may produce more accurate predictions than current state‐of‐the‐art ensemble approaches. Cui et al. [17] proposed a model that utilized a pre‐trained convolutional neural network (CNN) to extract emotional features from text. The architecture incorporated a reinforcement learning (RL) module, which included an RL selection layer to identify posts indicative of depression. Additionally, it employed a sentence‐level attention mechanism and a BiLSTM (bidirectional long short‐term memory) layer to capture contextual information. The outputs from both modules were then combined and passed to a classification model for depression detection. The model did, however, have several drawbacks. Because pre‐trained CNNs rely on characteristics learned from general datasets, they may not be able to properly capture the subtle emotional content unique to depression. Furthermore, choosing suitable reward functions is a critical decision that affects the performance of the RL component and can be difficult in real‐world social media scenarios. The presence of elaborate modules enhanced model complexity, resulting in increased training durations that may not be optimal for real‐world networks.

Tong et al. [18] proposed a cost‐sensitive pruning tree that included resampling weighted pruning for efficient feature extraction. The suggested pruning strategy was used to fine‐tune the Adaboost classifier, improving its generalization capabilities. To make their work more explainable, they adopted the TreeSHAP algorithm. However, there is a risk of adding bias through resampling and the additional complexity overhead associated with sophisticated resampling and weighted pruning processes, particularly for bigger datasets or more complex feature spaces.

In the study proposed by Shah et al. [19], a combination of different embeddings and features was experimented with, and the results were validated through classification using a BiLSTM model. However, BiLSTM models, while good at capturing sequential dependencies, can struggle with very long texts or complex patterns across different input sources, which may lead to reduced classification accuracy in such cases. The time complexity of the proposed method makes it suitable to scale in real‐time online social networks.

A multimodal deep‐learning method was suggested by Vandana et al. [20] with the goal of determining depression based on textual and auditory input from patients. Their method consisted of three stages: a textual CNN model, which trained a CNN solely on text characteristics; an audio CNN model, which trained a CNN solely on audio features; and a hybrid model, which included textual and audio data and included LSTM and Bi‐LSTM models. The authors proposed expanding their methods to online social networks (OSNs) as a future study.

## 4 RESEARCH GAP

Despite the significant strides in utilizing NLP and machine learning disorder classification from social media text [20, 21, 22, 23, 24], there are several conspicuous gaps in the current body of research. The following are the specific research gaps our study aimed to address:

1. Optimization techniques in model training: Many of the existing studies in this domain employ standard optimization techniques and overlook more innovative and resource‐efficient methods. Our research attempts to fill this gap by implementing several advanced optimization strategies such as gradient accumulation, adaptive learning rate scheduling, weight decay adaptation, and automated mixed precision (AMP) training. Additionally, we make use of Optuna for hyperparameters optimization. This comprehensive optimization strategy is designed to improve the efficiency of the training process and the performance of the models.
2. Employing an ensemble of transformer models: Another gap is the limited usage of ensemble learning in existing research. While single‐model approaches have been prevalent, there's a dearth of studies that harness the power of multiple transformer models. Our study addresses this gap by creating an ensemble of transformer‐ based models, including XLNet, RoBERTa, and ELECTRA, which can provide more robust and accurate predictions by leveraging their collective strengths. The collective power of multiple models can provide a more comprehensive understanding of longer posts, enhancing the ability to extract relevant features and detect depressive sentiments effectively.
3. Dynamic weight decay adjustment: Existing research largely implements a static approach to weight decay, which could potentially limit the models’ ability to generalize well to unseen data. Our study introduces dynamic weight decay adaptation, adjusting weight decay during training based on validation loss. This strategy is designed to further improve the models’ ability to generalize.

These gaps represent untapped potential in the existing literature. By addressing these gaps, our research seeks to contribute a more efficient and robust approach to the classification of mental health disorders from social media text data, enhancing the overall understanding and application of NLP and machine learning in the realm of mental health.

## 5 PROBLEM DEFINITION

The objective of this study is to formulate a solution for the problem of automatic classification of social media posts into fifteen different mental health categories. These categories are alcoholism, addiction, autism, ADHD, anxiety, bipolar disorder, BPD, eating disorder, depression, health anxiety, loneliness, PTSD, schizophrenia, social anxiety, and social watch [25].

This problem is a multi‐class text classification task, where each post can belong to one and only one category. It is assumed that each social media post is labelled with the correct mental health disorder.

In the broader perspective, this classification problem is inherently a task of natural language understanding (NLU), as it requires the understanding of the context and semantics of the social media posts to accurately categorize them. Moreover, the complexity of the problem is enhanced by the inherent noise in social media posts, which includes informal language, abbreviations, misspellings, and grammatical errors.

To tackle this problem, we employ transformer‐based models that have shown remarkable performance in various NLU tasks. Specifically, we use XLNet, RoBERTa, and ELECTRA models [26]. Each of these models is fine‐tuned on our dataset, and an ensemble of these models is used to make the final prediction.

The training process involves several steps, including hyperparameter tuning, which is guided by Bayesian Optimization using the Optuna framework [27]. During the training process, we employ a dynamic weight decay adjustment strategy, which is triggered by changes in the validation loss.

The final assessment of the model performance is done by applying various metrics, including accuracy, precision, recall, and F1‐score. These metrics are calculated based on the model's predictions on a separate test set.

Overall, the study provides a systematic approach to tackle the challenging tasks of automated classification of social media posts into various mental health categories. It not only contributes to the body of knowledge in text classification tasks but also opens avenues for potential applications in mental health monitoring and intervention.

## 6 METHODOLOGY

Our research methodology (as shown in Algorithm 1) is comprehensive, combining the strength of training‐based models like XLNet, RoBERTa, and ELECTRA with innovative and robust optimization strategies to categorize social media text into 15 categories of mental health disorders. The models were carefully modified to fit our task requirements and were fine‐tuned on our dataset. Here is an in‐depth look at our methodology:

ALGORITHM 1 **Pseudocode of the model training and evaluation**

**Table:** 

**Input**: Hyperparameters **Output**: Performance metrics *Step 1*: Initialize metrics and hyperparameters *Step 2*: Split the training data into train and validation data *Step 3*: Create DataLoader for train, validation and test data *Step 4*: Initialize the model, tokenizer, optimizer, and learning rate scheduler *Step 5*: **if** the model was interrupted and a checkpoint exists: **then** Load the model, tokenizer, optimizer, scheduler and scaler from the checkpoint Load the epoch and iteration numbers from where the training was interrupted Check and adjust the gradient accumulation steps if needed **end if** *Step 6*: Start training loop: **for** each epoch in total epochs: **do** **for** each batch in training data: **do** Perform forward propagation Perform backward propagation with gradient accumulation Update the parameters of the model Calculate validation loss Adjust weight decay based on validation loss **if** the validation loss is less than the previous best: **then** Reset early stopping counter **else** Increment early stopping counter **if** the counter reaches the limit, stop training: **then** Perform evaluation on validation data and calculate metrics **end if** **end if** **end for** **end for** *Step 7*: Save the state of the model if training is interrupted *Step 8*: Start evaluation loop on test data: Perform prediction on test data Calculate metrics Display confusion matrix and plot metrics Return model and calculated metrics

### 6.1 Data collection

Our data was sourced from the Reddit mental health dataset, an extensive and diverse compendium of social media posts collected from various mental health‐related forums on Reddit [28]. This choice of dataset is deliberate for a few reasons.

The Reddit mental health dataset is teeming with mental health‐related keywords and phrases, making it a highly relevant and rich resource for our research objective. The discourses within this dataset cover a broad spectrum of mental health disorders, offering valuable insights into the language and expressions associated with each disorder category.

Since the data is derived from real‐world interactions on Reddit, it retains the authenticity of user narratives and dialogues. The insights obtained from this data, therefore, reflect actual experiences and perceptions of mental health conditions.

The Reddit mental health dataset offers a large volume of data, which is crucial in training robust machine learning models. Further, the dataset is characterized by significant variability in terms of writing styles, expressions, and contexts, contributing to the diversity of data.

The significance of this dataset is amplified in the context of the COVID‐19 pandemic. It provides a timely snapshot of the heightened health anxieties and vulnerabilities that came to the forefront during this global crisis. This dataset allows us to capture and study these emergent nuances.

Our data collection process involved carefully extracting this data while maintaining data privacy standards. A crucial aspect of this process was ensuring a balanced representation of all 15 categories of mental health disorders. Our data collection strategy laid a solid foundation for the subsequent stages of our research.

**FIGURE 1:**
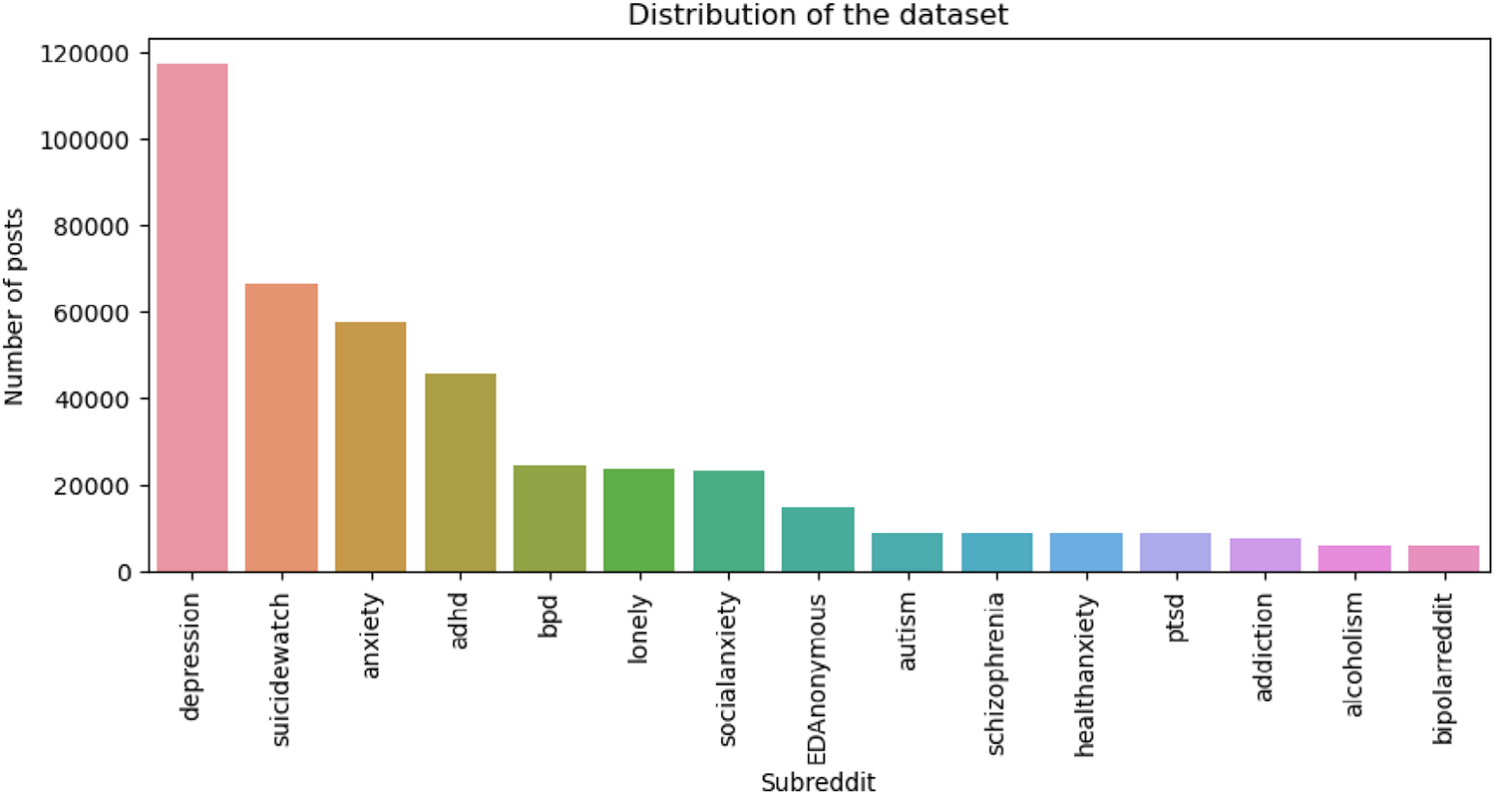
Distribution of dataset.

Figure 1 represents the distribution of the dataset across the 15 categories of mental health disorders.

The next step in our methodology, following data collection, is data processing, where we clean and transform the raw data to make it suitable for natural language processing (NLP) techniques. The data processing stage is critical for ensuring the quality and utility of the data that will be fed into our machine learning models.

### 6.2 Data processing

Data processing is a critical stage where the raw data is transformed and prepared for machine learning. Given that our raw data is inherently unstructured, filled with nuances of human language, processing it for computational models is a critical task. First, it involves cleaning the text data to remove irrelevant information. This includes removing special characters, numbers, and other non‐textual elements that might not contribute to our task of mental health disorder classification. The cleaning process also includes handling misspelled words and grammar inconsistencies.

Post‐cleaning, the text is tokenized, i.e. broken down into individual words or phrases. Tokenization is crucial in NLP as it helps convert a mass of unstructured text data into manageable, analysable pieces, often revealing interesting aspects of the text data.

The output of this phase is a processed dataset, ready to be ingested by our machine learning models for training.

### 6.3 Model training and configuration

The base models were instantiated with pre‐trained weights using the Hugging Face library. Pre‐training has revolutionized NLP by allowing models to learn from a massive amount of data beforehand. The models were then fine‐tuned on our dataset to align their learning to our specific task.

The dataset was split into a training set (80%) and a validation set (20%) via stratified sampling, which ensures a balanced distribution of labels in both sets. The training set is used for model learning, and the validation set is used for tuning the model parameters and validating the model's performance during training.

### 6.4 Gradient accumulation

Transformer‐based models like XLNet, RoBERTa, and ELECTRA are memory‐ intensive, and when dealing with large datasets, running these models might exceed the memory limit of the available hardware. Gradient accumulation (GA) helps overcome this limitation.

GA is a technique that allows us to handle large effective batch sizes while managing limited memory resources. It accumulates gradients over several mini‐batch updates before making an update in the backpropagation step.

### 6.5 Adaptive learning rate scheduling

Managing the learning rate effectively is critical to the convergence and overall performance of deep learning models. For this, we use the cosine annealing warm restarts scheduler (Equation (1)) [29]. The scheduler modifies the learning rate based on a cosine annealing schedule, which is a strategy that reduces the learning rate slower at the beginning and faster towards the end of a cycle.

(1) $${\mathrm{LR}}_{k}\;=\;{\mathrm{LR}}_{\min}\;+\;\frac{1}{2}\left({\mathrm{LR}}_{\max}\;-\;{\mathrm{LR}}_{\min}\right)\left(\;1\;+\;\cos\left(\;\frac{C_{\mathrm{curr}}}{C_{i}}\pi \right)\;\right)$$

where:

LR*_k_*: Learning rate at step *k*
LR_min_: Minimum learning rate
LR_max_: Maximum learning rate
*C*_curr_: Number of steps since the last restart
*C_i_*: Number of steps in the current restart period

This approach promotes better convergence and the overall performance of our models. The scheduler also employs periodic warm restarts, which reset the optimizer upon reaching a local minimum. This process encourages the model to explore different areas of the parameter space, further enhancing the model's performance.

### 6.6 Weight decay adaptation

Weight decay [30] is a regularization method used to prevent overfitting, which is a critical concern in machine learning models. We integrated weight decay into the AdamW optimizer, a process that curtails weights from growing too large by adding a fraction of the weight magnitudes to the loss function. The equation for the same is listed in Equation (2).

(2) $$\theta_{t+1}=\theta_{t}-\eta \left(\frac{{\hat{m}}_{t}}{\sqrt{{\hat{v}}_{t}}+\varepsilon }+\lambda \theta_{t}\right)$$

where:

*θ_t_*: Parameters at step *t*
*η*: Learning rate
${\hat{m}}_{t}$: Bias‐corrected first moment estimate (moving average of gradients)
${\hat{v}}_{t}$: Bias‐corrected second moment estimate (moving average of squared gradients)
*ε*: Small constant to avoid division by zero (usually set to 10^−8^)
*λ*: Weight decay coefficient

In AdamW, weight decay is applied explicitly through the term *λθ_t_*, decoupled from the gradient update $\frac{{\hat{m}}_{t}}{\sqrt{{\hat{v}}_{t}}+\varepsilon }$.

**Key points**:

- The term $\frac{{\hat{m}}_{t}}{\sqrt{{\hat{v}}_{t}}+\varepsilon }$ corresponds to the standard Adam update, based on the first and second moments of the gradients.
- The term *λ*θ_*t*_ is the explicit weight decay, applied directly to the parameters, reducing them in proportion to their current value.
- This decoupling makes AdamW more effective in applying regularization without interfering with the adaptive nature of Adam.

We enhanced this method by adjusting the weight decay rate based on the validation loss dynamically. If the validation loss improved from the previous epoch, we reduced the weight decay, and vice versa.

### 6.7 Automated mixed precision (AMP) training [31]

AMP training was used to expedite the training process and reduce its memory footprint. It allows the use of both single‐precision and half‐precision data types to optimize the performance of modern GPUs.

### 6.8 Early stopping

To prevent overfitting and maximize computational efficiency, we introduced an early stopping mechanism in our training process. Early stopping [32] is a form of regularization used to prevent overfitting when training a learner with an iterative method (as represented in Equation 3). It saves computational resources and prevents overfitting by stopping the training process if the performance on the validation set does not improve after a certain number of epochs.

Let *L*_val_(*e*) represent the validation loss at epoch *e*, and let *p* represent the patience (the number of epochs to wait for an improvement). The training process is stopped when:

(3) $$L_{\mathrm{val}}\left(e\right)\geq \min_{e^{\prime }\in \left[e-p,e\right]}L_{\mathrm{val}}\left(e^{\prime }\right)\;\mathrm{for}\;p\;\mathrm{consecutive}\;\mathrm{epochs}$$

**Explanation**:

- *L*_val_(*e*): Validation loss at epoch *e*.
- *p*: Number of consecutive epochs to wait without improvement (patience).
- $\min_{e^{\prime }\in \left[e-p,e\right]}\;L_{\mathrm{val}}\left(e^{\prime }\right)$: The minimum validation loss over the last *p* epochs.

If the validation loss doesn't improve for *p* consecutive epochs, early stopping is triggered, halting the training process to prevent overfitting and reduce computation. Our optimization with Optuna was adjusted to integrate early stopping. We implemented a pruning strategy that terminates unpromising trials at the early stages of the training process, enabling more efficient hyperparameter optimization.

### 6.9 Hyperparameters optimization

We employed Bayesian optimization with Optuna to find the optimal hyperparameters for our models. Optuna is a hyperparameter optimization framework that efficiently finds the best hyperparameters through a mix of exploration and exploitation. Its working is detailed in the sections below:

- Objective function: The objective function is designed to assess the model's performance based on specific hyperparameters, such as the accuracy in correctly identifying mental health conditions from social media content.
- Hyperparameter search space: The search space for hyperparameters includes:
  1. Learning rate: Influences the model's learning speed.
  2. Number of epochs: Number of complete passes through the training dataset.
  3. Gradient accumulation steps: Helps manage large batch sizes.
  4. Weight decay rate: Regularization parameter to prevent overfitting.
- Sampling strategy: Optuna employs a sampling strategy to select hyperparameters, balancing exploration (trying out new settings) and exploitation (refining known effective settings).
- Iteration: The model's performance on a validation dataset is used to assess each set of hyperparameters. Based on these evaluations, the probabilistic model is updated, which then informs future hyperparameter selections.
- Convergence: This process continues until the best‐performing hyperparameters are identified, which are then used to fine‐tune the transformer models.

#### 6.9.1 Fine‐tuning transformer models for mental health disorder detection

Once the optimal hyperparameters are determined, the individual transformer models (XLNet, RoBERTa, and ELECTRA) are fine‐tuned on a dataset consisting of social media posts labelled with 15 distinct mental health disorders.

Data preparation: The dataset is pre‐processed to clean the text data, including tokenization, removing irrelevant content (e.g. links, hashtags), and encoding the text into a format suitable for the models.

Model initialization: Each transformer model is initialized with pre‐trained weights.

Training: Each model is trained using the optimal hyperparameters. The training process includes:

1. Forward pass: The cleaned social media post data is fed into the model to generate predictions for the corresponding mental health disorder labels.
2. Loss calculation: The loss is calculated using a loss function (e.g. cross‐entropy loss) that compares the model's predictions against the true labels.
3. Backward pass: Gradients are computed and back‐propagated to update the model weights.

Validation: After training epochs, the model's performance is evaluated on a separate validation set to compute metrics such as accuracy, precision, recall, and F1 score specifically for the classification of mental health disorders.

The chosen hyperparameter ranges are tabulated in Table 1.

**TABLE 1:** Hyperparameter range.

Hyperparameters	Upper boundary	Learning rate
Learning rate	1 × 10^−6^	1 × 10^−4^
Epochs	2	10
GA steps	1	4
Weight decay	0.0	0.1

### 6.10 Ensemble learning

To leverage the strengths of individual models and enhance the overall performance, we employed an ensemble learning approach. We assembled an ensemble model using a voting classifier. This method integrates the strengths of the individual models, yielding a performance that is often superior to the best individual model. The following steps were employed in the designing the ensemble approach.

1. **Individual model (during training phase)**: Each transformer model (XLNet, RoBERTa, and ELECTRA) was fine‐tuned individually using the training dataset. Each model was subjected to Bayesian optimisation individually. This approach aided in determining the appropriate hyperparameters for maximising model performance. Following hyperparameter tweaking, each model was verified on a hold‐out validation set to guarantee that it performed optimally. Each model's performance (for example, accuracy and F1 score) was recorded and used later in the ensemble.
2. **Ensemble formation (during inference time)**: After the individual models were fine‐tuned and trained, the ensemble model was constructed using a voting classifier, a technique that aggregates the predictions of multiple models to make a final prediction. The voting approach combined soft and weighted voting and the entire process is illustrated below:
  - **Soft voting**: Each transformer model (XLNet, RoBERTa, and ELECTRA) provided a predicted class (mental health disorder) for a given social media post with different confidence scores. To ensure the model's confidence score reflected its true likelihood Platt scaling calibration technique was employed.
  - **Assigning weights**: Each model was assigned a weight based on its performance (e.g. accuracy, F1 score, validation set performance). Models with higher validation performance were given higher weights. For instance, when XLNet performed the best on the validation set, so it was assigned a weight of 0.5, while RoBERTa and ELECTRA were assigned weights of 0.3 and 0.2, respectively. The same is mathematically represented as shown in Equation (4).
(4) $$w_{i}=\frac{P_{i}}{\sum_{j=1}^{N}P_{j}}$$
    where:
*w_i_* is the weight assigned to model *i*,
*P_i_* is the performance score of model *i* (e.g. accuracy, F1 score, or validation set performance),
*N* is the total number of models,
$\sum_{j=1}^{N}P_{j}$ is the sum of the performance scores for all models, ensuring that the weights are normalized, such that:
(5) $$\sum_{i=1}^{N}w_{i}=1$$
  - **Combining probabilities and final prediction**: The probability scores from each model were weighted accordingly, and the weighted average of the probabilities was computed for each class. The class with the highest weighted average probability was selected as the final predicted class, combining both model confidence and relative weight based on performance. Equations (6) and (7) illustrates the same.
(6) $$P_{\mathrm{ensemble}}\left(y|x\right)=\sum_{i=1}^{N}w_{i}P_{i}\left(y|x\right)$$
    where:
*P*_ensemble_(*y|x*) is the weighted average probability for class *y* given input *x*,
*w_i_* is the weight assigned to model *i*,
*P_i_* (*y|x*) is the predicted probability of class *y* from model *i*,
*N* is the total number of models.
(7) $$y_{\mathrm{final}}=\arg\max_{y}P_{\mathrm{ensemble}}\left(y|x\right)$$

The use of soft voting in this scenario is acceptable since it takes into account not only the projected class but also the probability estimations (confidence levels) of each model's predictions. Soft voting makes more nuanced judgements by taking into account model confidence, particularly when predictions are vague or models differ in certainty. It mitigates individual model shortcomings by reducing the weight of low‐confidence predictions, allowing superior models to have a greater effect.

The weighted soft voting ensemble learning used here combines the strengths of individual models by giving more influence to high‐performing ones while still considering probability estimates from all models. This approach enhances prediction accuracy by reducing the impact of weaker models and adapting to each model's strengths across different classes or domains.

### 6.11 Model evaluation

After training, we evaluated our models using a separate test set. We computed several metrics, including accuracy, precision, recall, F1‐score, kappa, MCC, and error rate, to gauge the models’ performance comprehensively.

The evaluation phase is vital as it gives us an objective measure of how well our models have learned from the training data and how well they are likely to perform on unseen data.

Our methodology, featuring a blend of advanced optimization methods, serves to maximize the performance of our models on the mental health disorder classification task and to make efficient use of computational resources.

## 7 RESULT ANALYSIS

### 7.1 Model performance

In our study, we implemented an iterative process for model selection and hyperparameter optimization, leveraging 50 trials for each model—XLNet, RoBERTa, and ELECTRA. These models were then integrated into a voting classifier. Throughout these trials, we observed that RoBERTa consistently outperformed XLNet and ELECTRA in terms of various performance metrics, establishing it as the most suitable model for our specific task of mental health disorder classification from social media text data.

Table 2 displays the ideal hyperparameters for each model through the 50 trials.

**TABLE 2:** Ideal hyperparameters.

Model	Learning rate	Epochs	GA steps	Weight decay	Batch size	Loss function
XLNet	2.462 × 10^−5^	6	2	0.0802	8	Cross‐entropy
RoBERTa	1.806 × 10^−5^	6	3	0.0294	4	Cross‐entropy
ELECTRA	2.181 × 10^−5^	4	1	0.0542	8	Cross‐entropy

**FIGURE 2:**
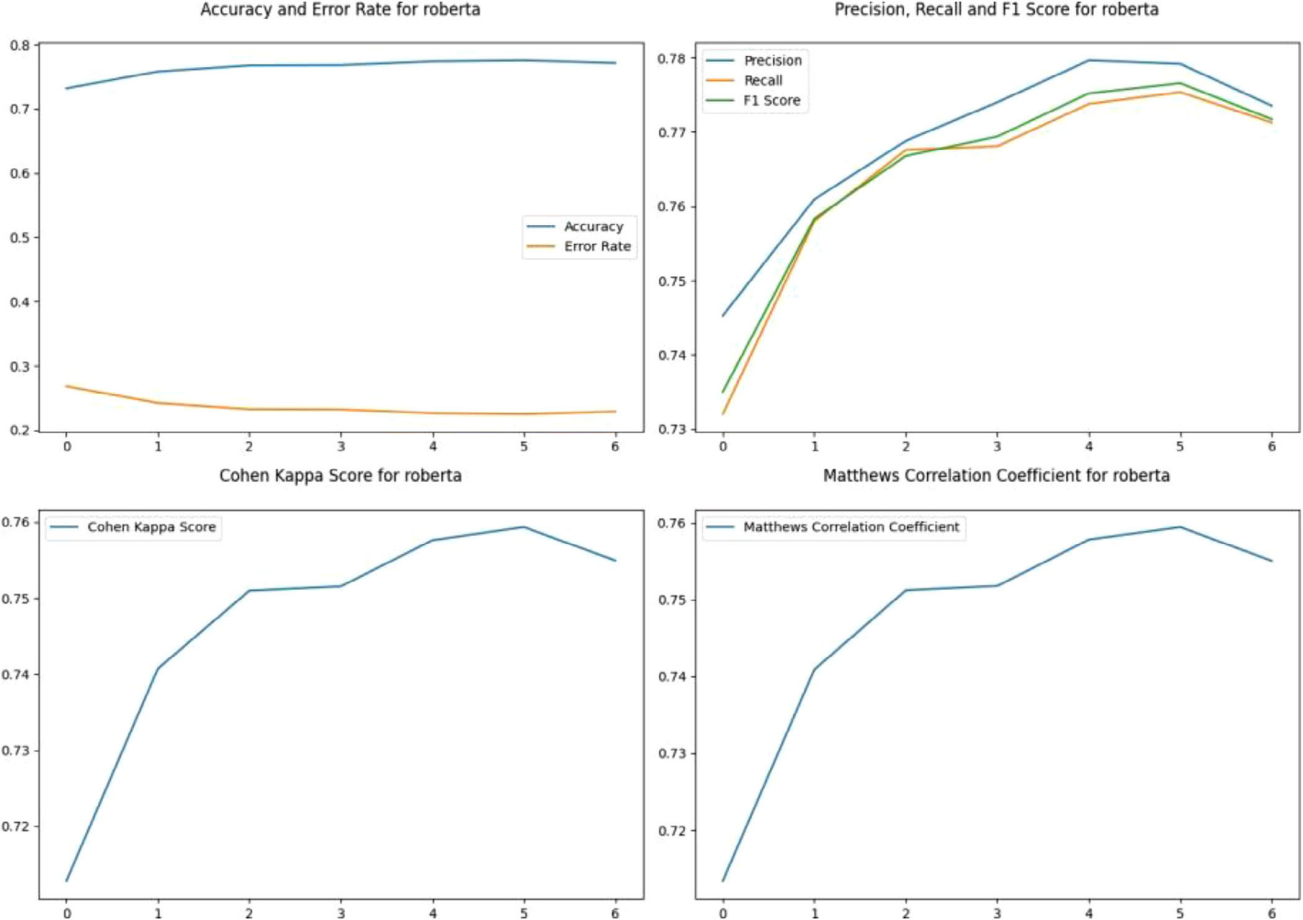
Performance metrics for RoBERTa.

**FIGURE 3:**
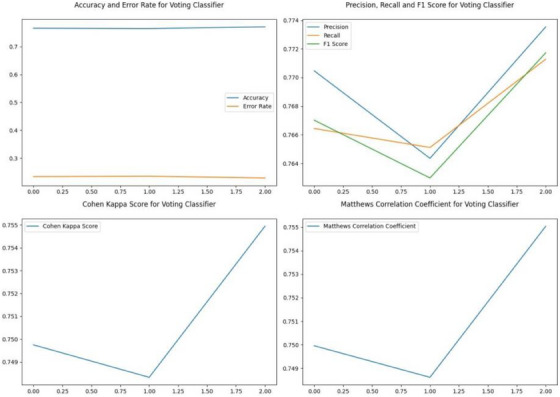
Performance metrics for the voting classifier.

Note: Here *n* is the number of parameters in a model, *m* is the number of input tokens and *k* is the number of models in the ensemble.

The voting classifier first tokenizes the text and converts it into input features that can be fed into the model. The input features are then passed to each model, and the model outputs the predicted class probabilities for each label. Once the predicted class probabilities for all models have been obtained, the method calculates the average predicted class probabilities across all models. Finally, the method chooses the label with the highest average probability as the final prediction. Figure 2 represents performance metrics of RoBERTa, the best performer among the three models alongside the Voting Classifier. Figure 3 represents performance metrics of the voting classifier.

While the optimized hyperparameters enhanced the model's performance, the presence of errors indicates room for further refinement, possibly through additional hyperparameter tuning or exploring alternative optimization strategies. The iterative trials and subsequent performance analysis underline the importance of thorough experimentation in achieving the most effective model for mental health disorder classification. The final results are tabulated in Table 3. The efficacy of the proposed method is compared against existing state of art relevant literature in Table 4.

**TABLE 3:** Model performances.

Model Name	XLNet	RoBERTa	ELECTRA	Voting Ensemble
Accuracy	0.767	0.775	0.755	0.780
Precision	0.772	0.774	0.768	0.781
Recall	0.767	0.772	0.755	0.780
F1‐score	0.766	0.766	0.737	0.779
Mathews correlation coefficient	0.568	0.75	0.66	0.78
Cohens kappa score	0.587	0.754	0.678	0.79
Inference time (ms)	50	45	40	55
Space complexity	O(n)	O(n)	O(n)	O(k.n)
Time complexity	O(m)	O(m)	O(m)	O(k.m)

**TABLE 4:** Performance metrics of various methods for mental health detection on social media.

Author(s) and year	Data set	Performance metrics (best achieved value)
Figuerêdo et al. [9] (2022)	eRisk (Reddit)	Proposed early fusion recall: 0.73, proposed late fusion precision: 0.76
Tavchioski et al. [10] (2023)	Reddit and Twitter	Averaging ensemble (RoBERTa, mental‐BERT, and BERTweet) on Reddit dataset accuracy: 0.592, averaging ensemble (RoBERTa and BERTweet) on Twitter dataset accuracy: 0.873
Lan et al. [11] (2024)	SWDD	Proposed method, DORIS Precision: 0.75
Shetty et al. [12] (2020)	Twitter	CNN Accuracy: 0.95, LSTM Accuracy: 0.93
Le‐Hinh et al. [13] (2023)	DepSign‐LT‐EDI@ACL2022	Proposed ensemble (BERT + RoBERTa + XLNet, RoBERTa + DepRoBERTa) accuracy: 66.8
Ogunleye et al. [14] (2024)	Social media datasets D1 (crawled from Reddit) and D2 (Reddit data prepared by authors)	Proposed SBERT + ensemble + Afinn F1 score for D1: 0.69, for D2: 0.76
Rizwan et al. [15] (2022)	Twitter tweets	ESG F1 score: 0.89
Liu and Shi [16] (2022)	Weibo	Proposed hybrid model accuracy: 90.27%
Cui et al. [17] (2022)	Multimodal depression data collected from Twitter	Accuracy of proposed model: 90.6%
Tong et al. [18] (2022)	Tsinghua Twitter depression dataset (TTDD) and CLPsych 2015 Twitter dataset (CLPsych2015)	TTDD dataset accuracy: 88.39%
Shah et al. [19] (2020)	eRisk 2017	Best performance F1 score: 0.81
Vandana et al. [20] (2023)	DAIC‐WOZ depression database	Hybrid BiLSTM precision: 0.75
**Proposed**	Reddit mental health dataset [28]	Proposed voting ensemble precision: 0.781, recall: 0.780

### 7.2 Word cloud heatmap analysis

The heatmaps for each label significantly contributed to our understanding of the most commonly used words and phrases in each mental health category. The heatmaps serve as a qualitative validation of our model's classification performance, as they align with our intuition and understanding of each disorder's characteristics.

**FIGURE 4:**
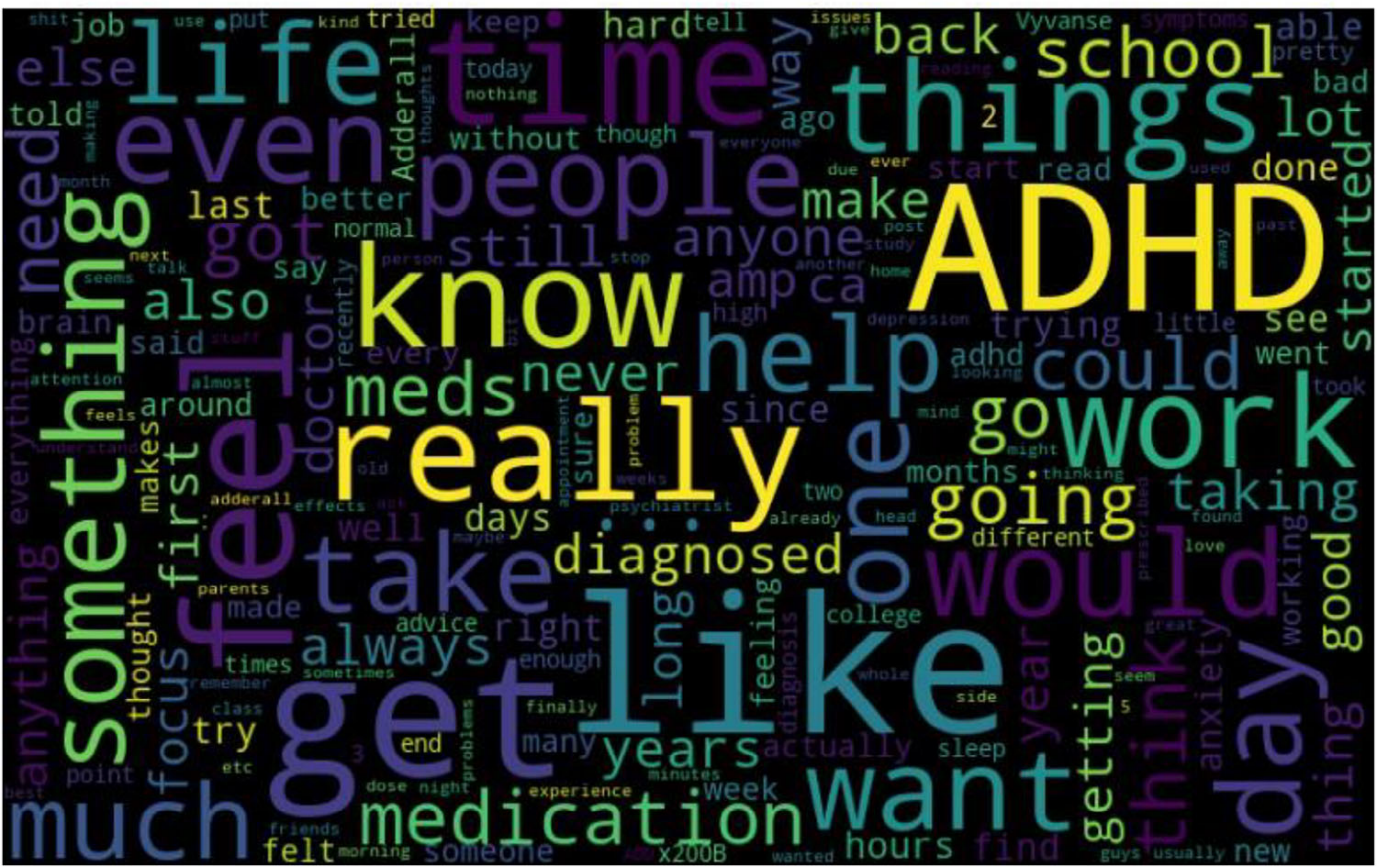
Word cloud for label: ADHD.

For instance, the word cloud for ‘ADHD’ as represented in Figure 4 has frequently occurring words like ‘focus’, ‘medication’, ‘time’.

**FIGURE 5:**
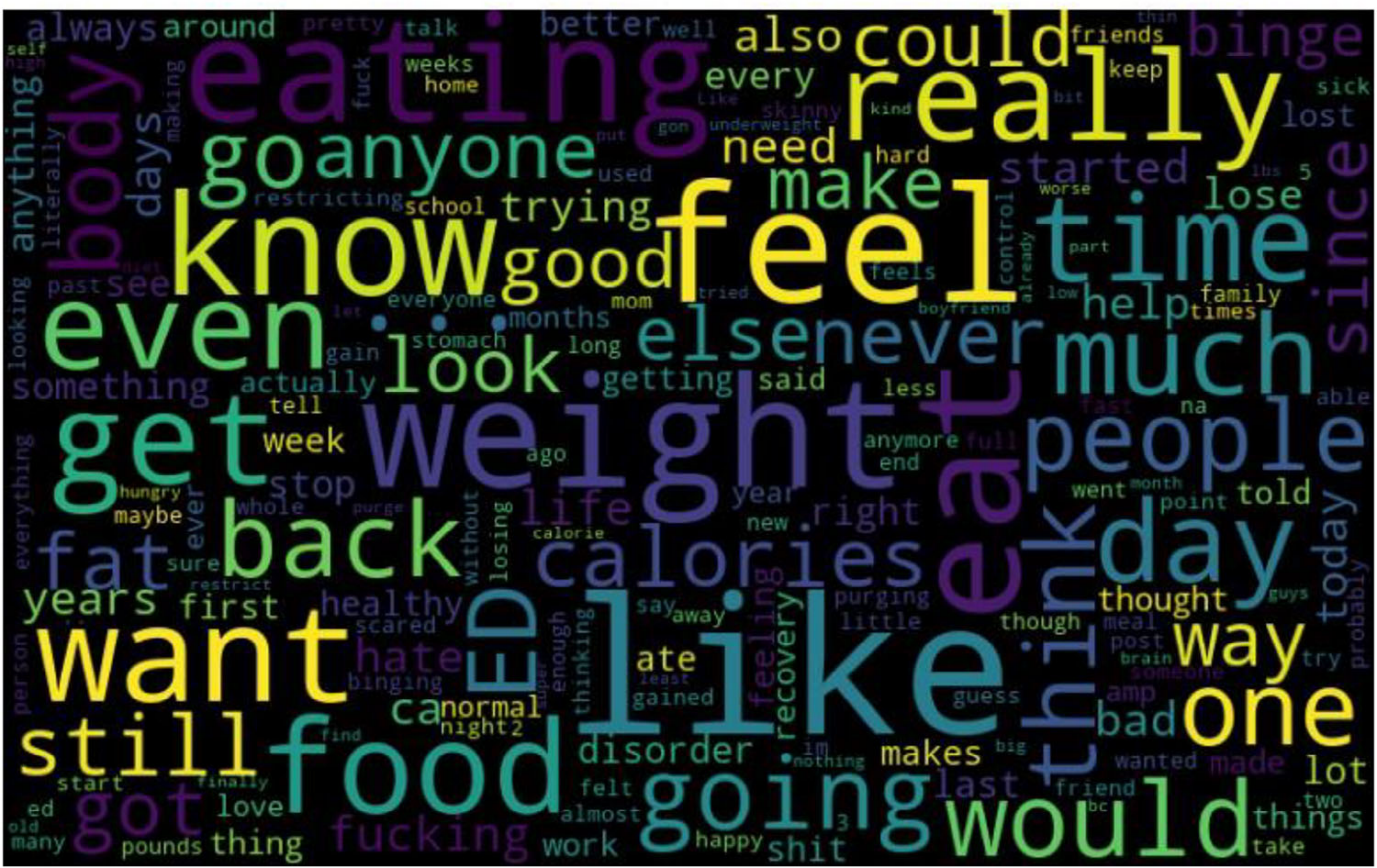
Word cloud for label: eating disorder.

In contrast, the word cloud for ’Eating Disorder’ as represented in Figure 5 highlights words like ‘weight’, ‘calories’, ‘food’, which coincide with our understanding of the concerns that someone with an eating disorder might have.

### 7.3 Emotion distribution analysis

Examining the distribution of different emotions (‘anger’, ‘anticipation’, ‘disgust’, ‘fear’, ‘joy’, ‘sadness’, ‘surprise’, ’trust’) across different mental health classes provides insightful observations.

**FIGURE 6:**
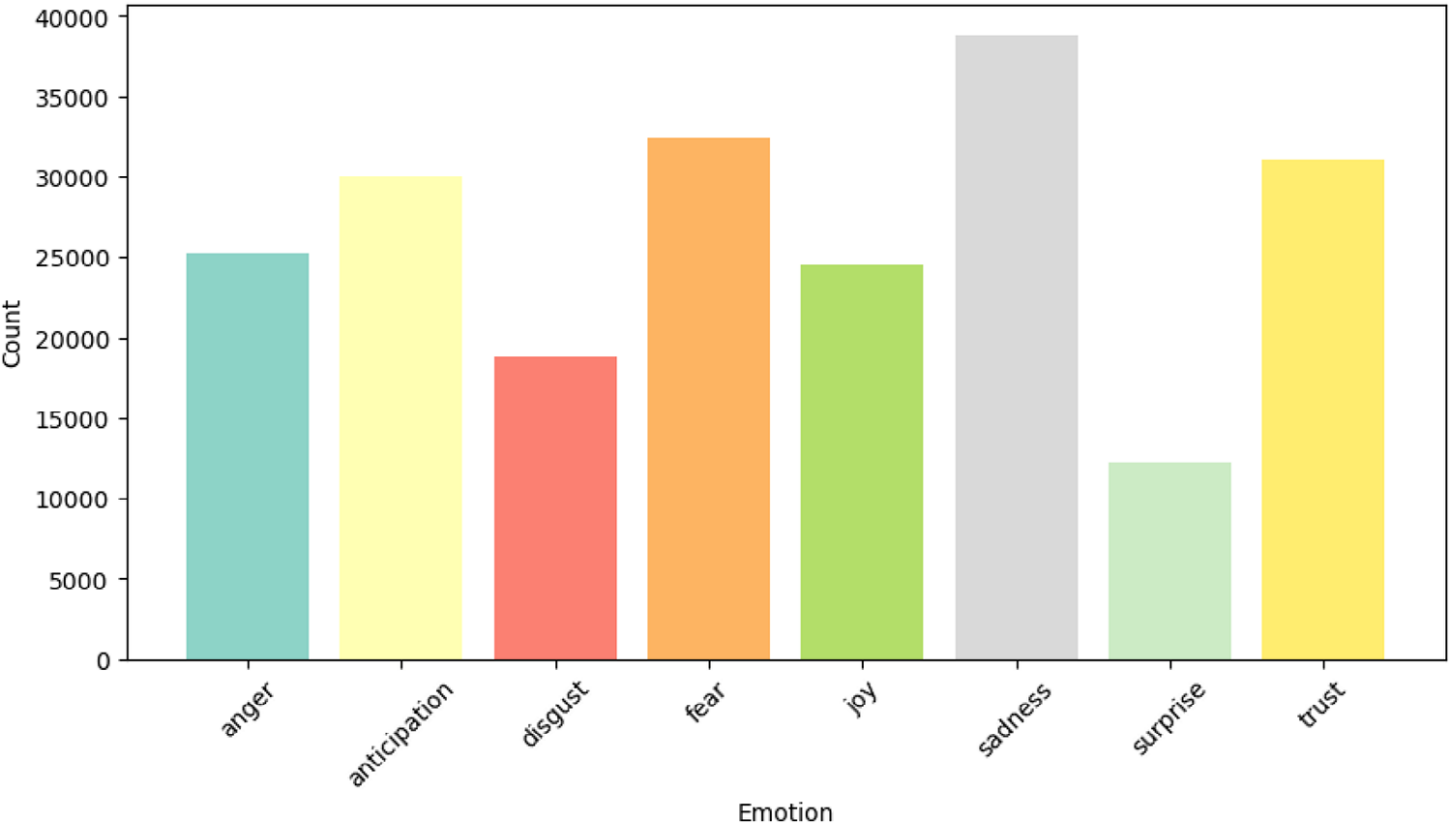
Distribution of different emotions in ‘depression’ posts.

A preliminary analysis of these emotion distribution plots could expose distinct emotional patterns linked with each mental health class. For instance, social media posts classified under ‘depression’ might display a higher prevalence of emotions such as ‘sadness’ and ‘fear’ while ‘joy’ might be less predominant as represented in Figure 6.

**FIGURE 7:**
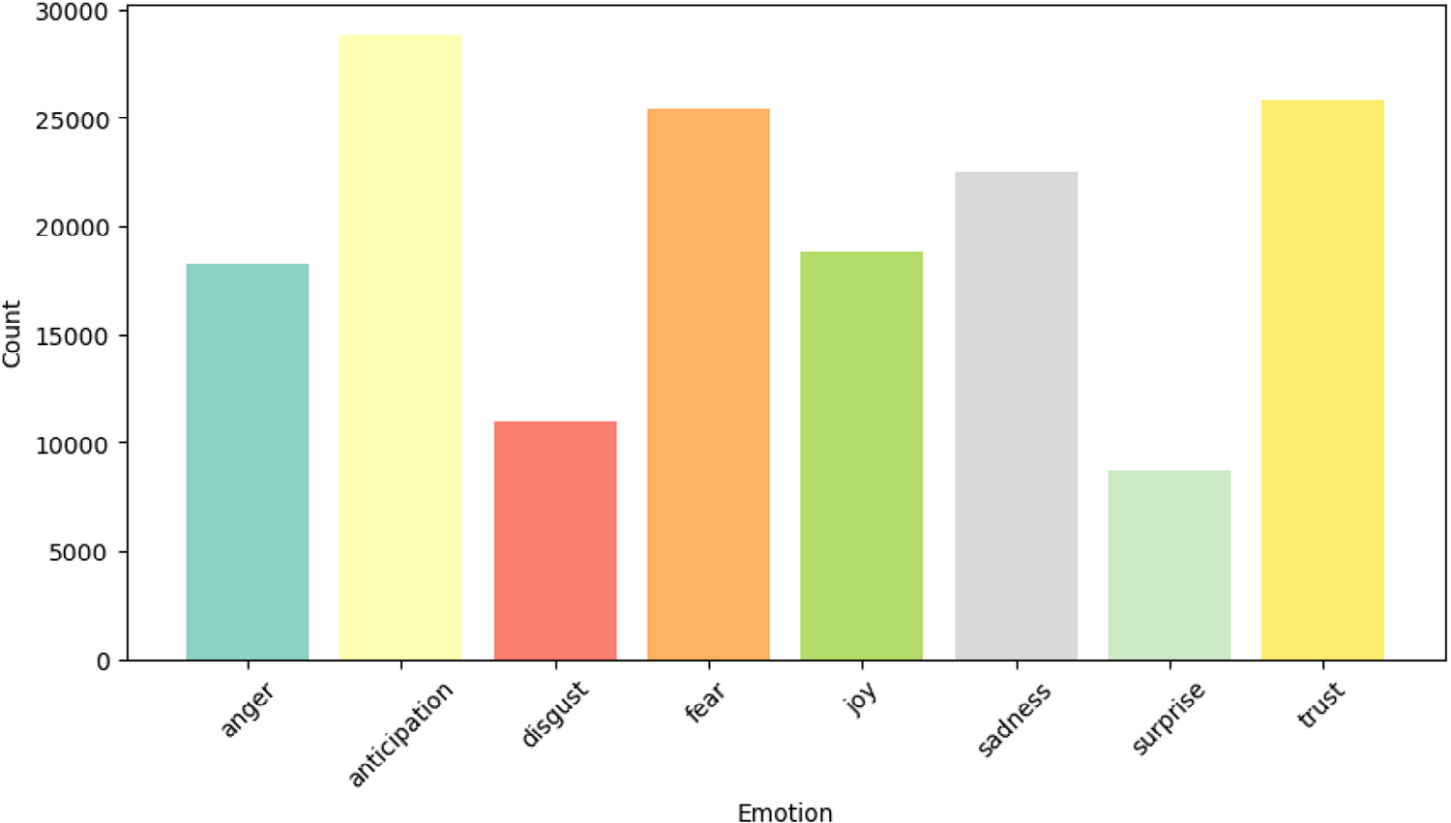
Distribution of different emotions in ‘social anxiety’ posts.

Conversely, posts classified under ‘health anxiety’ as represented in Figure 7 exhibit elevated levels of emotions like ‘fear’ and ‘anticipation’.

These patterns are derived using the “NRC Word‐Emotion Association Lexicon”, which assigns emotional scores to text based on a predefined lexicon [33]. This exploration not only contributes to our understanding of the emotional manifestation of different mental health disorders in text data but also aids in refining the categorization ability of our machine learning model. Consequently, understanding these emotional landscapes and incorporating them into the model could lead to improved performance in future mental health disorder classification tasks.

## 8 DISCUSSION

Our study aimed at employing a selection of the most proficient transformer‐based models and advanced optimization techniques to identify and classify mental health disorders based on social media text data. We examined XLNet, RoBERTa, and ELECTRA models, fine‐tuned them to our task, and optimized their performance using several strategies, including gradient accumulation, adaptive learning rate scheduling, weight decay adaptation, automated mixed precision (AMP) training, and ensemble learning.

In the process of fine‐tuning the models, despite the impressive performance of RoBERTa, the voting classifier was able to slightly outperform it. This was done by weighted averaging the predicted class probabilities across the models and choosing the label with the highest average probability as the final prediction. Nevertheless, the presence of errors indicates an avenue for potential improvement and scope for further refinement.

The importance of meticulously optimized hyperparameters was underlined through iterative trials and performance evaluation. The results from the Bayesian optimization conducted using Optuna allowed us to identify the most suitable hyper‐ parameters for each model. The improvement in model performance with these hyperparameters further emphasized their significance in machine learning tasks.

The ensemble model demonstrated superior performance compared to the individual models, achieving higher accuracy, precision (0.781), recall (0.780), and F1‐score (0.779). This indicates the ensemble model is more robust and reliable in predicting mental health disorders, as it effectively balances precision and recall. Additionally, the high Mathews correlation coefficient (0.78) and Cohen's kappa score (0.79) suggest the ensemble model offers better reliability and agreement in classification than the individual models. The ensemble model's high precision and recall scores (both over 0.780) show it can accurately identify true positives without compromising its ability to capture all relevant instances, which is crucial for mental health disorder classification. Among the individual models, ELECTRA demonstrated slightly lower performance, particularly in its F1‐score (0.737), suggesting it struggled more with balancing precision and recall compared to XLNet and RoBERTa.

Although the ensemble model performed better, its inference time was 55 ms, which was a little bit longer than that of the individual models, which ranged from 40 ms (ELECTRA) to 50 ms (XLNet). This is a small computational trade‐off for the improvement in performance, as the ensemble adds more complexity by fusing together different models. In addition, the ensemble's space and temporal complexity is greater than that of the individual models (O(k.n) and O(k.m) versus O(n) and O(m)), which suggests the need for more processing power, especially in situations where real‐time applications are being used.

Transformer models excel at comprehending context and utilize the significant words as anchors for classification. The repetition of particular words in the text gives extra context for transformers to grasp the primary themes of each disorder. The voting ensemble, consequently, benefits from this, as different models may concentrate on various aspects (keywords, emotional tone), and merging their outputs enhances the overall classification.

The proposed voting ensemble approach combines the unique strengths of multiple language models, including XLNet, RoBERTa, and ELECTRA, to create a robust and adaptable classifier for mental health disorder classification. XLNet excels at understanding the broader context across multiple sentences, which is crucial when discussing mental health symptoms. RoBERTa's focus on subtle word associations and semantic relationships enhances the ensemble by accurately identifying nuanced emotional expressions. ELECTRA's generator–discriminator framework adds precision by detecting word‐level anomalies and shifts in tone, which are common in social media posts. Together, these complementary models provide a well‐rounded classifier that can capture both broad and minute patterns in social media posts, making it highly effective for real‐world mental health applications where emotional expression is diverse and complex.

The application of the Word Cloud heatmap facilitated a qualitative assessment of our model's performance. It confirmed that the most frequently used words and phrases in each mental health category accurately aligned with the characteristic expressions associated with the respective disorders. This alignment demonstrated the effectiveness of our model in recognizing and classifying mental health disorders based on text data. The heatmaps for each mental health label serve as a qualitative validation method, showing which words or emotions are most closely associated with each disorder. This supports the explainability of the transformer models, providing insights into why a post was classified in a certain way. If the heatmap for depression posts shows high intensity for words like ‘hopeless’ or ‘alone,’ and the model also detects high levels of sadness and fear in the post, this is consistent with the expected emotional profile of someone experiencing depression. Such qualitative validation ensures that the models are correctly identifying the relevant features for each mental health disorder, leading to more accurate and reliable results in practice. Furthermore, these heatmaps reinforce the voting ensemble's decisions by aligning the output of individual models with meaningful patterns in the data. When multiple models in the ensemble recognize similar emotional patterns or key phrases, the ensemble can make a more confident classification, contributing to the higher accuracy observed. The word clouds and keyword patterns also assist in model interpretability, allowing researchers to validate that the models are focusing on the right terms, which correspond to domain knowledge about each mental health condition.

The emotion distribution analysis allowed us to observe unique emotional patterns associated with different mental health classes. This in‐depth examination of emotions within text data, utilizing the NRC Word‐Emotion Association Lexicon, not only enriched our understanding of how various mental health disorders manifest in written language but also suggested the potential for refinement of the model's categorization capability. The emotional patterns in the text provide crucial contextual clues that transformer models like XLNet, RoBERTa, and ELECTRA use to analyse the text. These models’ attention mechanisms can focus on the relevant emotional words or phrases, enhancing their ability to identify the underlying mental health condition. When the voting ensemble combines the predictions from these models, it enhances the classification accuracy by combining their strengths in detecting these emotion‐related patterns. For example, if one model identifies a high presence of fear and sadness in a post, while another model detects low levels of joy, the voting ensemble can combine these predictions, increasing the likelihood of correctly classifying the post as related to depression.

### 8.1 Study limitations

The study admits various limitations that may have an influence on the methods utilised. First, fine‐tuning three transformer models on a dataset of social media postings labelled with 15 mental health illnesses cannot accurately represent the disorders’ intricacy and multifarious character, resulting in misclassification. Furthermore, the use of a static dataset implies that significant contextual details like tone, irony, and cultural allusions can get overlooked, which is critical for effectively analysing social media content. While the use of Bayesian optimization for hyperparameter tweaking attempts to improve model performance, it can jeopardise the interpretability of the ensemble method. The voting ensemble technique enhances overall accuracy over individual models, but it can conceal the explanations behind specific predictions, which is critical in the complicated field of mental health. Finally, the necessity for regular dataset updates to capture the temporal dynamics of social media trends is emphasised, since the models may become less successful in reflecting current mental health discourse over time if these changes are not made. These limitations highlight the need for more study to improve the robustness and application of prediction models in this field.

## 9 CONCLUSION AND FUTURE WORK

Our study demonstrates the potential of advanced machine learning techniques, specifically transformer‐based models, in mental health disorder classification based on social media data. By applying XLNet, RoBERTa, and ELECTRA models to the Reddit Mental Health Dataset, we achieved promising results in identifying 15 distinct mental health conditions. The hyperparameter optimization through Bayesian optimization further enhanced the models’ performance, substantiating its significance in achieving computationally efficient and accurate results.

The successful implementation of these models reflects the value of incorporating AI and NLP techniques into mental health research. Our results indicate that with the right tools, social media platforms can serve as rich datasets for mental health analysis. They offer a glimpse into public sentiment and emotional states in real time, allowing researchers to track mental health trends, highlight emerging concerns, and aid in early intervention strategies.

Looking ahead, we envision numerous directions for future research. The proposed work concentrates on Reddit, but mental health dialogues also take place on other social media sites like Twitter, Instagram, and TikTok. Future research could compare mental health patterns across these platforms by creating a cross‐platform dataset. This would offer a complete overview of mental health trends and enable the models to generalize more effectively by considering variations in language style, content length, and platform culture. While our research is focused on the English language, it is conceivable that similar techniques could be applied to other languages. This could expand the understanding of mental health discourse on a global scale, potentially illuminating cultural or regional differences in mental health experiences and discussions. Refining transformer models like mBERT or XLM‐R can help investigate mental health topics across diverse linguistic groups and regions, providing insights into how mental health is discussed worldwide. This knowledge can also assist in developing intervention strategies that are tailored to specific cultural contexts. However, as we utilize personal discussions shared online, we must remain cautious and respectful of individual privacy. The need for automated data analysis tools that allow models to learn from decentralized data (such as on users’ devices) without directly accessing sensitive information, promoting ethical data usage is the need of the hour.

Future research should focus on incorporating temporal analysis to better understand the dynamic nature of mental health states as reflected in social media posts. This could involve implementing time series analysis techniques and exploring recurrent models like LSTMs combined with transformers to predict the progression of mental health disorders. By tracking sentiment evolution over time, researchers could identify patterns that may signal impending crises or improvements in mental well‐being. Additionally, developing methods for dynamic risk assessment, detecting seasonal and cyclic patterns, and integrating multi‐modal temporal data could enhance the accuracy and applicability of mental health analysis models. Furthermore, in order to facilitate early predictive therapies, future models may be trained on continual personal social media data in order to identify early warning indicators and offer a more comprehensive picture of how mental health problems change over time.

While our study does not venture into practical implementation, we believe our findings can be used to develop practical tools for mental health professionals, policymakers, and researchers. For instance, our models could be integrated into a real‐time monitoring system on social media platforms, alerting professionals to emerging mental health trends or potential crises. Combining behavioural data from social media with diagnostic tools such as the DSM‐5 or ICD‐10 criteria for mental health disorders could help validate the models’ predictions and enhance their utility in clinical practice. Furthermore, our work may inspire new approaches in the field of natural language processing (NLP). The lessons learned from our hyperparameter optimization process can be applied to other NLP tasks, enhancing model performance in a broad range of applications. Future research might focus on increasing the efficiency of the ensemble technique. Transformers are computationally costly; thus, research on model compression approaches like as pruning, quantization, or knowledge distillation might assist minimize computational costs and make models more accessible for real‐time applications, particularly for organizations with limited resources.

In conclusion, our study underscores the potential of leveraging AI in understanding and addressing mental health issues, a global concern that demands innovative solutions. Through a thoughtful combination of cutting‐edge AI techniques, rich social media data, and a commitment to ethical research, we believe our work takes a meaningful step towards improving mental health awareness, understanding, and intervention.

## AUTHOR CONTRIBUTIONS

**Nisha P. Shetty**: Methodology; project administration; supervision; writing—review & editing. **Yashraj Singh**: Conceptualization; formal analysis; methodology; supervision; validation; visualization. **Veeraj Hegde**: Supervision; validation. **D. Cenitta**: Supervision. **Dhruthi K**.: Writing—review and editing.

## CONFLICT OF INTEREST STATEMENT

The authors declare no conflicts of interest.

## Data Availability

Data is available from corresponding author upon request.
